# Physicochemical, microbiological, and microstructural changes in germinated wheat grain

**DOI:** 10.1371/journal.pone.0331620

**Published:** 2025-09-09

**Authors:** Darigash Shaimerdenova, Aigul Omaraliyeva, Baltash Tarabayev, Zhanar Chakanova, Damira Iskakova, Gaini Sarbassova, Maigul Kizatova, Sandugash Anuarbekova

**Affiliations:** 1 Department of Science, LLP “Research and Production Enterprise “Innovator”, Astana, Kazakhstan; 2 Technology of Food and Processing Industries, Saken Seifullin Kazakh Agrotechnical University, Kazakhstan; 3 Department of Pharmaceutical Technology, Kazakh National Medical University, Almaty, Kazakhstan; Institute of Genetics and Developmental Biology Chinese Academy of Sciences, CHINA

## Abstract

This study investigates the physicochemical, microbiological, and microstructural changes in soft wheat grain during germination under varying moisture conditions: moderately dry, moist, and wet. Pre-harvest sprouting can severely compromise grain quality and usability; however, understanding germination-induced changes offers insights into potential utilization strategies. Physical parameters—including thousand-kernel weight, test weight, and falling number—showed strong correlation with germination time, decreasing by 8.2%, 22%, and 74%, respectively. Microstructural analyses using optical microscopy, scanning electron microscopy (SEM), and Raman spectroscopy revealed substantial degradation of starch granule morphology and kernel structure, with compact vitreous endosperm becoming porous and disorganized as germination progressed. To optimize germination conditions for technological application, a central composite design with three factors (moisture, temperature, and time) was employed, analyzed using Statgraphics Centurion 19. Response surface modeling identified optimal conditions for starch content (22% moisture, 31°C, 84 h), protein content (21% moisture, 30°C, 72 h), and minimal microbial contamination (14% moisture, 33°C, 8 h). These findings provide a foundation for processing germinated soft wheat grain into value-added products, even when exposed to unfavorable harvest conditions.

## Introduction

**Fig 1:**
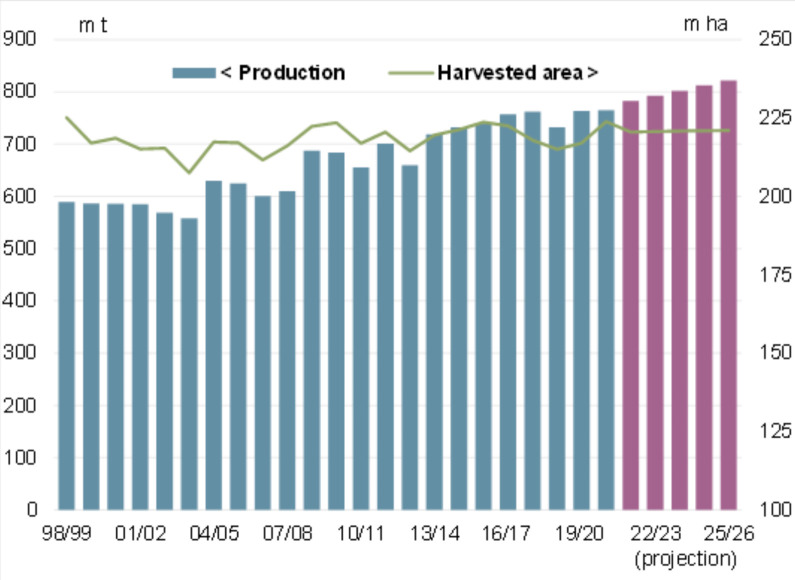
Wheat: Global production and harvested area.

Soft wheat (*Triticum aestivum* L.) is one of the most important cereal crops globally, including in Kazakhstan [1,2]. According to the International Grains Council, wheat remains a staple commodity in international trade, occupying the largest sowing area worldwide—220 million hectares [3] (Fig 1). However, wheat production is highly susceptible to climatic variability [4–7].

High humidity and temperature conditions often trigger natural pre-harvest sprouting (PHS) in cereal crops, reducing grain quality and causing significant economic losses worldwide [5,8,9]. PHS is particularly likely in wheat-growing regions experiencing rainfall during and after grain maturation, such as Canada, the United States, Australia, South Africa, Central Asia, Western Europe, and the northern regions [10]. In Kazakhstan, up to 40% of soft wheat harvested in 2023 was classified as non-standard due to PHS [11], with global losses estimated at $1.2 billion annually [10]. The increasing frequency of extreme weather events and projected climate shifts are expected to exacerbate PHS [12]. Heavy rainfall during grain filling induces early germination, diminishing yield and adversely affecting technological quality [13,14]. These climate-driven declines in grain quality are part of a wider pattern in Kazakhstan’s agricultural sector, where maintaining productivity in both crop and livestock systems increasingly depends on the implementation of biotechnological solutions [15–17]. Understanding the biochemical and structural changes in germinated grain is critical for exploring potential valorization strategies. Previous research has shown that starch is the most affected component during wheat germination [18], primarily due to increased α-amylase activity, which degrades starch and reduces grain usability. This enzymatic degradation can lower yield by up to 30% and may render wheat unsuitable for human consumption [18]. The falling number (FN), which reflects α-amylase activity, is a key indicator of germination extent, strongly correlated with the proportion of sprouted kernels [19]. Germination triggers extensive biochemical transformations, particularly hydrolysis of starch and other macromolecules, due to activation of α- and β-amylases [20]. These processes influence both food and industrial applications. For instance, sprouted wheat starch has been explored for producing oleogels [21], biofilms [22], fiber materials [23], and cyclodextrins [13]—each with potential commercial applications.

Despite advances, there remains a lack of integrative studies addressing the physicochemical, microbiological, and microstructural changes in germinated wheat. Most existing work focuses either on enzymatic or structural aspects, often under simplified or single-factor conditions [9,24]. Additionally, microbiological safety concerns of germinated grain under shifting climatic conditions are underexplored [25,26].

Given these gaps, the present study aims to investigate the physicochemical, microstructural, and microbiological changes occurring in soft wheat grain germinated under controlled laboratory conditions. The findings are intended to inform strategies for the effective utilization of sprouted wheat grain, particularly in regions where pre-harvest sprouting is increasingly prevalent.

## Materials and methods

### Materials

Soft wheat grain of the “Tәuelsizdik” variety, harvested in 2024 and cultivated in the Karagandy region of Kazakhstan, was used for this study. The grain exhibited the characteristics summarized in Table 1.

**Table 1:** Characteristics of materials used in the study.

Parameters	Wheat Grain “Tәuelsizdik” Variety
Protein content,%	14.44 ± 0.05
Fat content,%	2.07 ± 0.02
Starch content,%	57.48 ± 0.03
Moisture content,%	13.36 ± 0.02

The data are presented as mean ± SE (standard error); the sample size was three (n = 3), and the level of statistical significance was set at ≤ 0.05.

### Germination

The wheat grain utilized was germinated according to protocol described by Ahmed, Ragab [27]. In brief, the grain samples were rinsed with potable water, followed by surface sterilization using a 1% NaCl solution for 30 minutes. After disinfection, the samples were thoroughly washed with water until a neutral pH was achieved. Subsequently, the grains were subjected to hydration in a 1:2 grain-to-water ratio at 20°C for 24 hours. Post-soaking, the grains were evenly distributed in germination trays, covered with a moistened white cotton cloth, and allowed to incubate at 25 ± 1°C for 24, 48, or 72 hours. These samples were used for the determination of thousand-kernel weight, falling number, and test weight.

For assessment of physicochemical, microbiological, and microstructural transformations, the soaked grain samples were pre-dried to target moisture contents of 15.5%, 18.0%, and 20.5%, representing three standardized grain storage moisture levels as defined in regulatory guidelines [28]. The samples were placed in germination trays and incubated for 7.7 to 88.3 hours at 16.6–33.4 ± 1°C and 85% relative humidity, following the experimental design. The germinated samples were then dried in a convection oven at 50–55°C for 5 hours to achieve a final moisture of 9.0–12.0%.

### Flour preparation

The germinated wheat was ground to a particle size below 0.8 mm using a Stegler LM-500 at 28,000 rpm. The produced flour was then kept in sealed glass vessels at ~4°C to maintain sample stability for subsequent testing.

### Determination of physical condition indicators

The falling number (FN) of the flour was measured following the Hagberg–Perten method as outlined in ISO 3093 [29], employing a PCHP-7 instrument with a cooling system. The analysis was conducted using 7 g of flour (standardized to 14% moisture) dispersed in 25 mL of distilled water.

The thousand-kernel weight was assessed in accordance with the international standard ISO 520:2010 [30]. Grain test weight was determined following ISO 7971–3:2019 [31].

### Determination of physicochemical parameters

Moisture content in all samples, as well as protein levels in both the grain and resulting flour, were determined according to standard protocols established by the American Association of Cereal Chemists (AACC) [32]. Starch content was measured following the ISO method [33].

### Determination of microbiological properties

Isolation of microorganisms from wheat grain was carried out using a protocol described by Patil and Kukade [34]. To do so, 10 g of grain was placed in a porcelain mortar with 90 mL of sterile physiological saline. The grain was ground using a sterile porcelain pestle under aseptic conditions. Serial dilutions were prepared from the initial suspension by adding 1 mL of the homogenate into 9 mL of sterile saline, constituting the first tenfold dilution (10 ⁻ ¹). Titration continued sequentially up to a dilution of 10 ⁻ ¹⁰, using a new pipette for each step to prevent cross-contamination and ensure data accuracy. From each dilution, 0.1 mL was plated onto selective nutrient media. Plates containing meat peptone agar (MPA) were incubated aerobically at 30 ± 1°C for 18–24 hours to assess total aerobic mesophilic bacteria. To quantify yeast and filamentous fungi, aliquots of the final dilutions were inoculated onto Sabouraud dextrose agar and incubated at 25 ± 1°C for five days. All culture media were obtained from HiMedia Laboratories Pvt. Ltd., India.

Analyses were performed in triplicate to ensure reproducibility and calculate mean values. Qualitative assessment of the microbiota was conducted based on morphological and cultural characteristics. Gram staining was used for bacterial smear identification.

Fungal taxa were primarily identified based on morphological descriptors [35]. Microscopic characteristics were examined using prepared slides viewed under a Biolab 6T trinocular biological microscope (China), equipped with planachromatic lenses at magnifications of 40 × , 100 × , and 1000 × . Samples were taken from both ungerminated and germinated wheat grain.

### Scanning electron microscopy (SEM)

The surface morphology of germinated wheat grain samples was examined using a Quanta 3D 200i scanning electron microscope (FEI, USA). Germinated and lyophilized wheat kernels were sectioned with a razor blade to obtain transverse cross-sections. These sections were then mounted onto aluminum stubs using carbon adhesive tape. Imaging was conducted at accelerating voltages of 10 kV and 5 kV. SEM micrographs of the cross-sections were captured at magnifications of 1000 × , 2500 × , and 5000 × .

### Raman spectroscopy analysis

Raman spectral measurements were performed using a Solver Spectrum Raman spectrometer (NT-MDT, Germany) equipped with a 473 nm excitation laser. Signal acquisition was conducted with a 600/600 diffraction grating, providing a spectral resolution of 4 cm ⁻ ¹. Sample preparation involved bisecting a wheat kernel transversely and mounting it vertically with the fresh cross-section facing upward. The laser beam was focused on the sample using a 100 × objective lens, creating a spot approximately 2 µm in diameter. The signal accumulation time was set to 200 seconds. For each sample, ten spectra were recorded from different positions on the cross-section to assess potential heterogeneity in composition and structural features.

### Optical microscopy imaging

To visualize the microstructure, an optical microscope DM 6000M (Leica, Germany) was employed using 5× and 10 × objective lenses in dark-field mode. Due to the uneven surface of the grain sections, focus bracketing was applied—a technique involving the merging of a series of images captured at different focal depths to enhance overall depth of field and image clarity.

## Data analysis

The findings are reported as the mean values of three independent measurements, accompanied by standard deviation. Experimental data analysis was carried out using Microsoft Excel and MathCad. All results were evaluated at a 95% confidence interval. Statistical significance was assessed through two- ANOVA. The investigation of the combined effects of wheat grain germination parameters on chemical composition indicators and microbial contamination levels was conducted using a central composite design with three factors (2³ + star) [36]. Data analysis and response surface modeling were performed using the Statgraphics Centurion 19.

The optimization process targeted three key response variables that characterize the grain’s quality: starch content, protein content, and microbial contamination levels. The selected independent (controllable) variables included moisture content (x₁), temperature (x₂), and germination time (x₃). Experimental planning was based on a full factorial design, with each factor varied at two levels. All other experimental conditions were held constant. The outcome of each trial was evaluated based on changes in the following response variables: starch content (y₁), protein content (y₂), and total microbial load (y₃). The experimental design incorporated three independent variables and consisted of 16 runs, including two center points. The degrees of freedom associated with the residual error were six. The coding and decoding of factor levels are presented in Table 2.

**Table 2:** Decoded values of experimental factors.

Experimental Factors	Coded values	*X*_*min*_	*X*_*i0*_	*X*_*max*_	*∆X*
Moisture, %	*х*_*1*_	15,5	18	20,5	2,5
Temperature,°С	*х*_*2*_	20	25	30	5
Time, h	*х*_*3*_	24	48	72	24

## Results

### Alterations in the physical properties of soft wheat grain during germination

**Fig 2:**
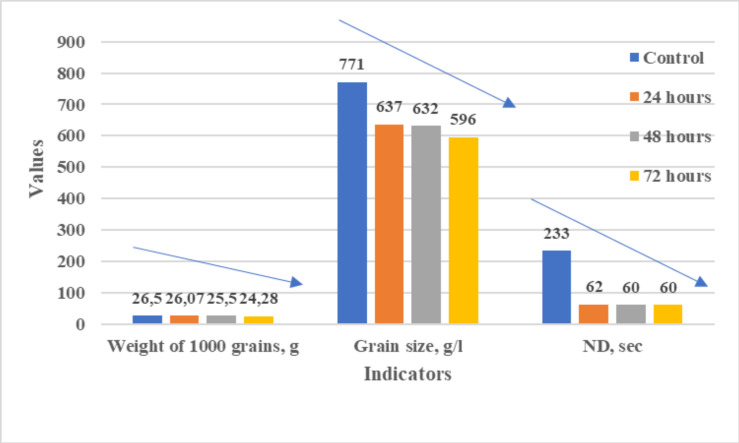
Changes in the physical properties of soft wheat grain. The data are presented as mean values ± standard error (SE); the sample size was three, and the significance level was set at ≤ 0.05.

To evaluate the impact of germination on the physical properties of the grain, thousand-kernel weight, test weight, and falling number were measured. The analysis of germinated soft wheat grain revealed a direct correlation between germination duration and the deterioration of physical properties. Thousand-kernel weight declined from 26.52 g to 24.28 g (−8.2%), while test weight decreased from 771 g/L to 596 g/L (−22%). The most pronounced reduction was observed in the falling number, which dropped from 233 seconds to 60 seconds, indicating a 74% decline (Fig 2).

### Three-factor experiment

As a result of the conducted experiments, values for starch content, protein content, and microbial contamination were obtained under varying conditions of moisture, temperature, and germination time for soft wheat grain of the “Tәuelsizdik” variety (Table 3).

**Table 3:** Results of the three-factor experiment using soft wheat of the Tәuelsizdik variety.

Experiment No	Levels of Controlled Factors						Starch Content, %	Protein Content, %	Microbial Load, CFU/g
	Coded values			Truel values					
	*х*_*1*_	*х*_*2*_	*х*_*3*_	Moisture, %	Temperature,°С	Time, h	*у*_*1*_	*у*_*2*_	*у*_*3*_
1	0	0	0	18	25	48	68.62 ± 0.77	14.26 ± 0.15	10^10^
2	−1	−1	0	15.5	20	24	66.13 ± 0.63	12.71 ± 0.16	10^9^
3	+1	−1	0	20.5	20	24	67.28 ± 0.79	13.4 ± 0.09	10^10^
4	−1	+1	0	15.5	30	24	67.26 ± 0.68	13.42 ± 0.17	10^9^
5	+1	+1	0	20.5	30	24	68.83 ± 0.55	14.06 ± 0.06	10^10^
6	−1	0	−1	15.5	20	72	67.31 ± 0.61	13.43 ± 0.12	10^10^
7	+1	0	−1	20.5	20	72	68.79 ± 0.83	14.07 ± 0.05	10^9^
8	0	0	0	15.5	30	72	68.63 ± 0.64	14.06 ± 0.15	10^9^
9	−1	0	+1	20.5	30	72	70.02 ± 0.81	14.75 ± 0.11	10^10^
10	+1	0	+1	13.79	25	48	67.24 ± 0.52	13.63 ± 0.14	10^8^
11	0	−1	−1	22.20	25	48	68.79 ± 0.52	14.46 ± 0.10	10^10^
12	0	+1	−1	18	16.59	48	67.03 ± 0.71	13.62 ± 0.09	10^9^
13	0	−1	+1	18	33.41	48	68.32 ± 0,88	14.47 ± 0.11	10^8^
14	0	+1	+1	18	25	7.64	67.04 ± 0.59	13.65 ± 0.20	10^10^
15	0	0	0	18	25	88.36	68.77 ± 0.64	14.43 ± 0.09	10^10^

The data are presented as mean values ± standard error (SE); the sample size was three, and the significance level was set at ≤ 0.05. The visualizations of the experiments conducted are provided in the supplementary materials S1 Fig.

### Effect of germination on starch content

In the conducted study, starch content ranged from 66.13% to 70.02%.

Analysis of variance for starch content (Table 4) revealed that three effects had P-values below 0.05, indicating that they were statistically significant at the 95.0% confidence level.

**Table 4:** Analysis of variance for starch content.

Values	Sum of Squares	Degrees of Freedom (df)	Mean Square	F-Ratio	p-Value
*х*_*1*_	4,91966	1	4,91966	43,83	0,0006
*х*_*2*_	4,00918	1	4,00918	35,72	0,0010
*х*_*3*_	4,87502	1	4,87502	43,43	0,0006
*х*_*1*_^*2*^	0,197035	1	0,197035	1,76	0,2334
*х*_*1*_ *х*_*2*_	0,0136125	1	0,0136125	0,12	0,7395
*х*_*1*_ *х*_*3*_	0,0028125	1	0,0028125	0,03	0,8794
*х*_*2*_^*2*^	0,655717	1	0,655717	5,84	0,0521
*х*_*2*_ *х*_*3*_	0,0021125	1	0,0021125	0,02	0,8954
*х*_*3*_^*2*^	0,316133	1	0,316133	2,82	0,1443
Residual	0,673462	6	0,112244		
Total	15,213	15			

In this table, the analysis of variance partitions the variability in starch content into individual components corresponding to each effect. It then evaluates the statistical significance of these effects by comparing their mean squares to the estimated experimental error. In this case, three effects exhibit P-values less than 0.05, indicating that they are significantly different from zero at the 95.0% confidence level. The model fitting results for starch content, along with the regression coefficients, are presented in Table 5.

**Table 5:** Regression coefficients.

Coefficients	Values
Constant	46,9618
*х*_*1*_	0,982597
*х*_*2*_	0,587551
*х*_*3*_	0,0534429
*х*_*1*_^*2*^	−0,0233339
*х*_*1*_ *х*_*2*_	0,0033
*х*_*1*_ *х*_*3*_	0,0003125
*х*_*2*_^*2*^	−0,0106418
*х*_*2*_ *х*_*3*_	−0,000135417
*х*_*3*_^*2*^	−0,000320709

**Fig 3:**
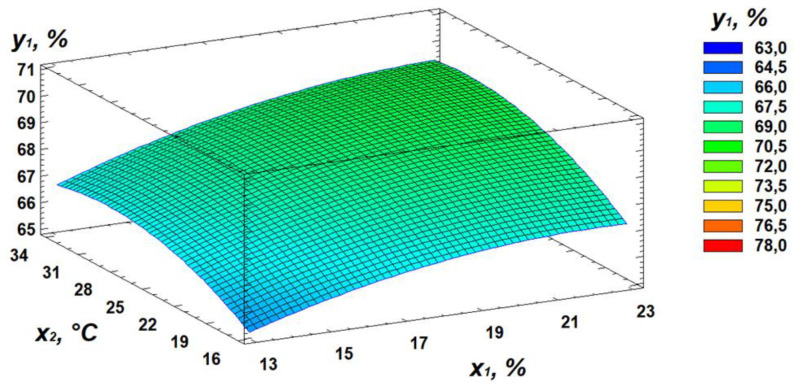
Response surface plot showing the effect of moisture and temperature on starch content.

**Fig 4:**
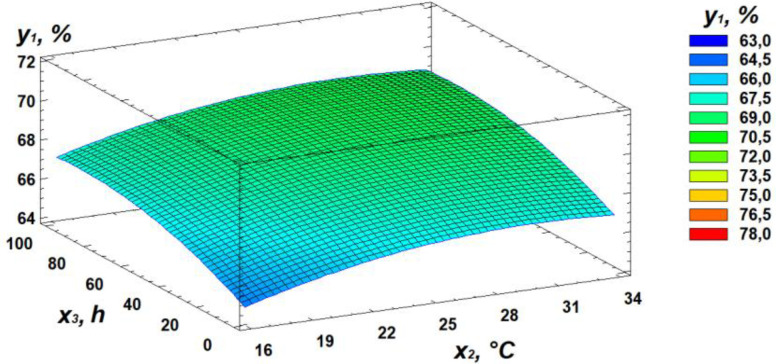
Response surface plot showing the effect of temperature and germination time on starch content.

**Fig 5:**
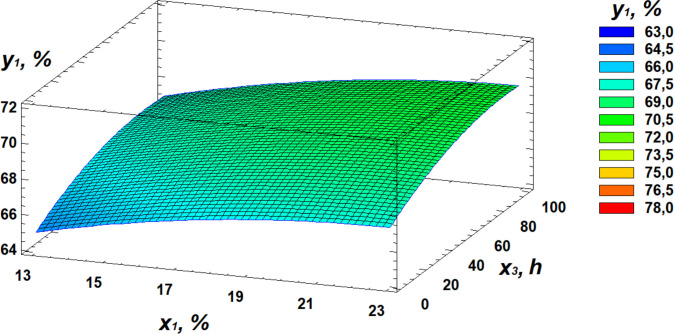
Response surface plot showing the effect of moisture and germination time on starch content.


Based on regression equation (1), three-dimensional surface models were constructed to represent the relationship between starch content and the germination parameters of the wheat grain (Figs 3–5).

$$\begin{array}{c} {\text{y}}_{1}=46,9618+0,982597\;{\text{ x}}_{1}+0,587551\;{\text{ x}}_{2}+0,0534429\;{\text{ x}}_{3}-0,0233339\;{{\text{x}}_{\text{1}}}^{2}+0,0033\;{\text{ x}}_{1}\;{\text{ x}}_{2}\\ +\;0,0003125\;{\text{ x}}_{1}\;{\text{ x}}_{3}-\;0,0106418\;{{\text{x}}_{\text{2}}}^{2}-\;0,000135417\;{\text{ x}}_{2}\;{\text{ x}}_{3}-\;0,000320709\;{{\text{x}}_{\text{3}}}^{2} \end{array}$$ (1)

Analysis of the generated response surface plots indicated that the optimal starch content is achieved at a moisture level of 22%, a temperature of 31°C, and a germination time of 84 hours.

### Effect of germination on protein content

In the conducted study, protein content ranged from 12.71% to 14.75%. Based on the experimental results, a relationship between protein content and the germination parameters of the wheat grain was established and demonstrated by the regression equation (2):


$$\begin{array}{c} {\text{y}}_{2}=-\text{1},\text{28697 }+\text{ 0},\text{920016 }{\text{ x}}_{1}+\text{ 0},\text{341384 }{\text{ x}}_{2}+\text{ 0},\text{0373418 }{\text{ x}}_{3}-\text{ 0},\text{0222562 }{{\text{x}}_{\text{1}}}^{2}\\ -\text{ 0},\text{00556404 }{{\text{x}}_{\text{2}}}^{2}-\text{0},\text{0000625 }{\text{ x}}_{2}\;{\text{ x}}_{3}-\text{0},\text{000244567 }{{\text{x}}_{\text{3}}}^{2} \end{array}$$ (2)

**Fig 6:**
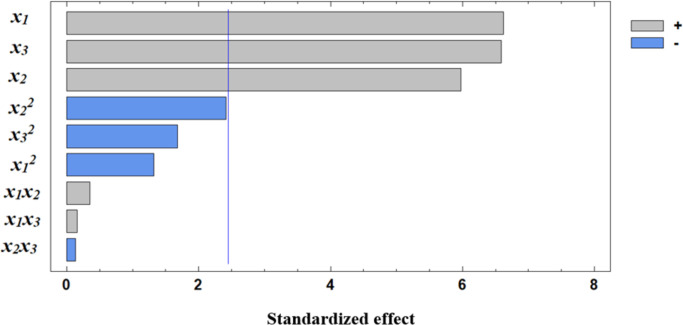
Standardized effects of independent factors on protein content.

Based on the derived regression equation, three-dimensional surface models were constructed to illustrate the relationship between protein content and the germination parameters of the wheat grain. A visual representation of the contribution of individual independent variables is provided by the Pareto chart of standardized effects (Fig 6). The statistical significance of the regression equations for protein content and microbial contamination is presented in the supplementary materials (S1 Table).

**Fig 7:**
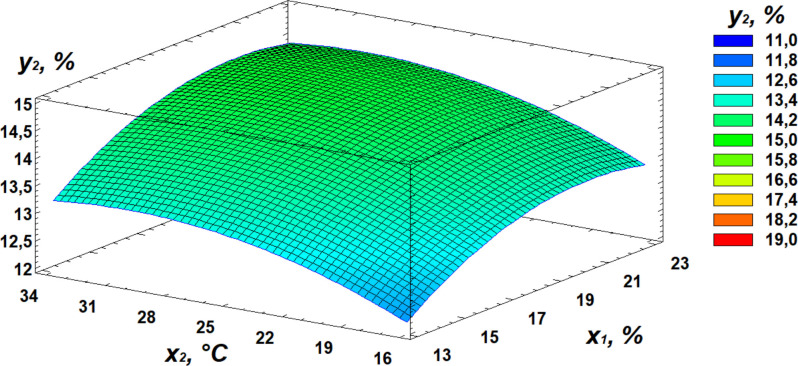
Response surface plot showing the effect of moisture and temperature on protein content.

**Fig 8:**
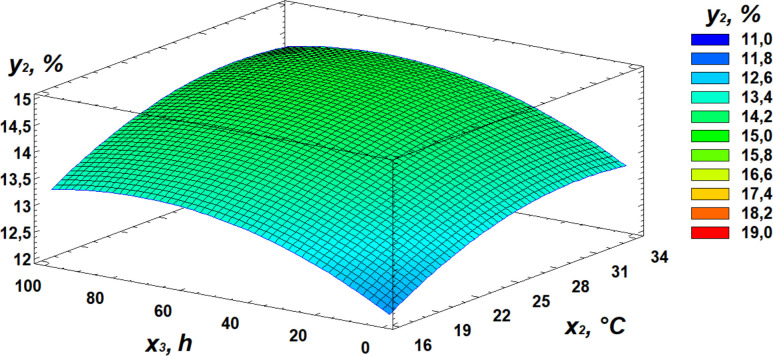
Response surface plot showing the effect of temperature and germination time on protein content.

**Fig 9:**
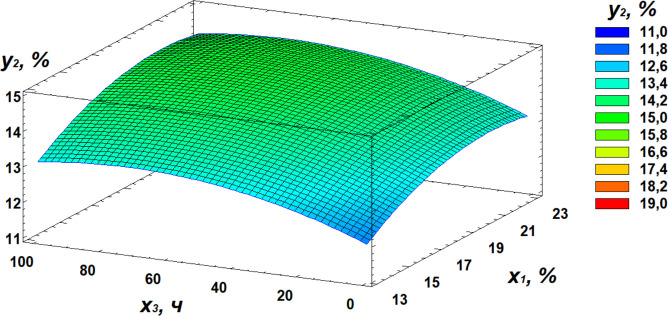
Response surface plot showing the effect of moisture and germination time on protein content.

Analysis of the Pareto chart indicates that germination temperature has the most significant positive effect on protein content. Further clarification of the effects of independent variables can be obtained through response surface analysis, represented by 3D plots of the observed dependencies (Figs 7–9).

Analysis of the obtained data revealed that the optimal conditions for maximizing protein content are as follows: moisture level of 21%, temperature of 30°C, and germination time of 72 hours.

### Effect of germination on microbial load of wheat grain

During germination, the microbial load of wheat grain ranged from 10⁸ to 10¹⁰ CFU/g and is described by the following regression equation (3):


$$\begin{array}{c} {\text{y}}_{3}=-\text{1},\text{22}\times \text{1011}+\text{7},\text{6}\times \text{109 }{\text{ x}}_{1}+\text{ 3},\text{14}\times \text{109 }{\text{ x}}_{2}+\text{ 6},\text{52}\times \text{108 }{\text{ x}}_{3}-\text{ 2},\text{58}\\ \;\;\;\times \text{108 }{{\text{x}}_{\text{1}}}^{2}+\text{ 1},\text{8}\times \text{108 }{\text{ x}}_{1}\;{\text{ x}}_{2}-\text{ 3},\text{75}\times \text{107 }{\text{ x}}_{1}\;{\text{ x}}_{3}-\text{1},\text{28}\times \text{108 }{{\text{x}}_{\text{2}}}^{2}+\text{ 1},\text{53}\times \text{10}-\text{5 }{\text{ x}}_{2}\;{\text{ x}}_{3}+\text{ 2},\text{39}\times \text{105 }{{\text{x}}_{\text{3}}}^{2} \end{array}$$ (3)

**Fig 10:**
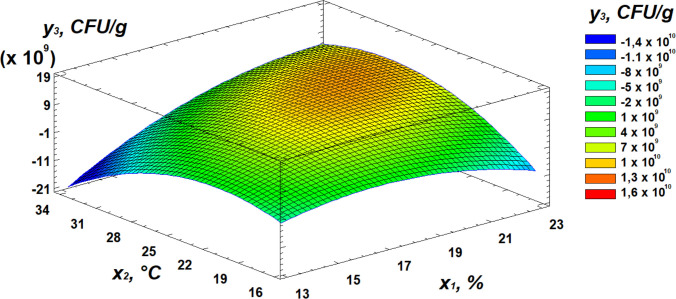
Response surface plot showing the effect of moisture and temperature on total microbial contamination.

**Fig 11:**
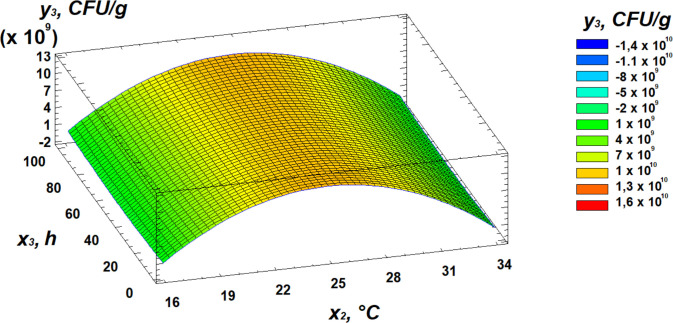
Response surface plot showing the effect of temperature and germination time on total microbial contamination.

**Fig 12:**
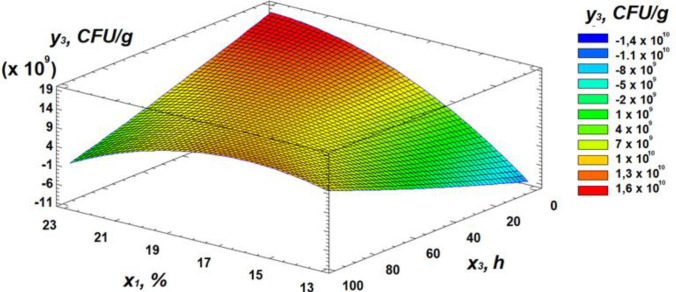
Response surface plot showing the effect of moisture and germination time on total microbial contamination.

Analysis of the Pareto chart indicates that temperature exerts the strongest negative effect on total microbial contamination, while moisture has the most significant positive influence. Further insights into the effects of the independent variables can be obtained from the response surface analysis, presented as 3D surface plots in Figs 10–12.

Analysis of the resulting response surface indicated that the optimal conditions for minimizing total microbial contamination in flour are as follows: 14% moisture content, 33°C temperature, and 8 hours of germination.

**Fig 13:**
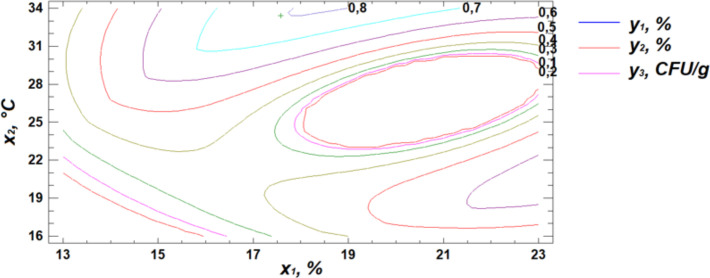
Response surface cross-section projections showing the effect of moisture and temperature on starch content, protein content, and total microbial contamination.

**Fig 14:**
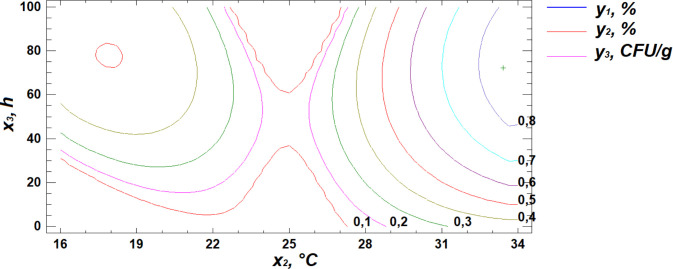
Response surface cross-section projections showing the effect of temperature and germination time on starch content, protein content, and total microbial contamination.

**Fig 15:**
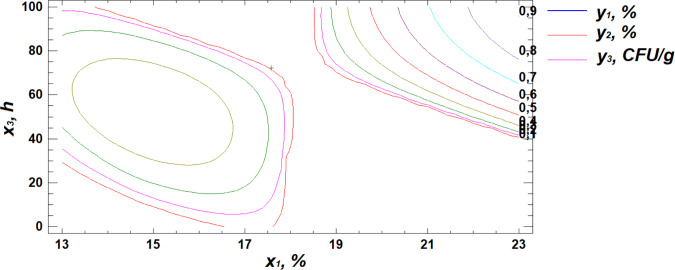
Response surface cross-section projections showing the effect of moisture and germination time on starch content, protein content, and total microbial contamination.

To identify the optimal combination of germination parameters for all three response variables—starch content, protein content, and total microbial contamination—a multiple response optimization was performed. This approach allows for the simultaneous optimization of several outcomes by maximizing the desirability function (Figs 13–15).

The obtained response surface cross-section projections, illustrating the relationship between the response variables (y) and the controlled factors (x), made it possible to determine the combination of factor levels that maximize the desirability function within the defined optimal region (Table 6). This combination represents the specific conditions under which the optimal outcome is achieved.

**Table 6:** Optimization of soft wheat grain germination process based on the desirability function.

Factors	Min.	Max.	Optimal Value	Response	Optimized Responce
Moisture, %	13,8	22,2	17,5	Starch Content, %	69,0
Temperature,°С	16,6	33,4	33,4	Protein Content, %	14,5
Time, hours	7,6	88,4	72,3	Total Microbial Load, CFU/g	1010

Thus, the obtained results enabled the identification of optimal germination parameters for soft wheat grain through the application of the developed mathematical model.

### Microbiological characterization of wheat grain during germination

Since microbial populations are predominantly located in the outer layers of the grain and within the germ, the samples were ground prior to analysis. Bacteriological examination of 16 samples of *T*ә*uelsizdik* wheat grain—both untreated and germinated under various moisture, temperature, and time conditions—revealed (Table 7) extensive microbial colonization. The presence of both bacterial and fungal contamination was detected across the samples.

**Table 7:** Microbiological indicators of wheat grain (in Triplicate).

Sample ID	Growth on MPA, CFU/g				Growth on Sabouraud Agar, CFU/g			
	1	2	3	Mean	1	2	3	Mean
Initial (ungerminated)	100 × 10^6^	100 × 10^6^	100 × 10^4^	100 × 10^5^	100 × 10^10^	100 × 10^10^	100 × 10^10^	100 × 10^10^
1	10^8^	10^8^	10^8^	10^8^	10^10^	10^10^	10^10^	10^10^
2	10^9^	10^8^	10^8^	10^8^	10^10^	10^9^	40 × 10^11^	13 × 10^10^
3	10^10^	10^10^	10^10^	10^10^	10^10^	10^10^	10^10^	10^10^
4	10^10^	10^10^	10^10^	10^10^	10^10^	10^9^	10^6^	10^8^
5	10^10^	10^10^	10^10^	10^10^	10^10^	10^10^	10^10^	10^10^
6	10^10^	10^10^	10^10^	10^10^	10^9^	10^10^	10^10^	10^10^
7	10^10^	10^9^	10^7^	10^9^	10^10^	10^10^	10^10^	10^10^
8	10^10^	10^8^	10^6^	10^8^	10^10^	10^10^	10^10^	10^10^
9	10^10^	10^10^	10^10^	10^10^	10^10^	10^10^	10^10^	10^10^
10	10^10^	10^6^	10^6^	10^7^	10^10^	10^10^	10^10^	10^10^
11	10^10^	10^10^	10^10^	10^10^	10^10^	10^10^	10^10^	10^10^
12	10^7^	10^10^	10^10^	10^9^	10^10^	10^10^	10^10^	10^10^
13	10^7^	10^8^	10^8^	10^8^	10^10^	10^10^	10^10^	10^10^
14	10^10^	10^10^	10^10^	10^10^	10^10^	10^10^	10^10^	10^10^
15	10^10^	10^10^	10^10^	10^10^	10^10^	10^10^	10^10^	10^10^

In the control sample, bacterial counts were within the range of 10 × 10⁶ CFU/g, while filamentous fungi reached levels of approximately 10 × 10¹¹ CFU/g. Subsequently, bacterial counts increased significantly compared to the control, rising by 5–6 orders of magnitude, with the majority of samples exhibiting concentrations around 10¹⁰ CFU/g. Fungal contamination was also detected at a level of 10¹⁰ CFU/g.

**Fig 16:**
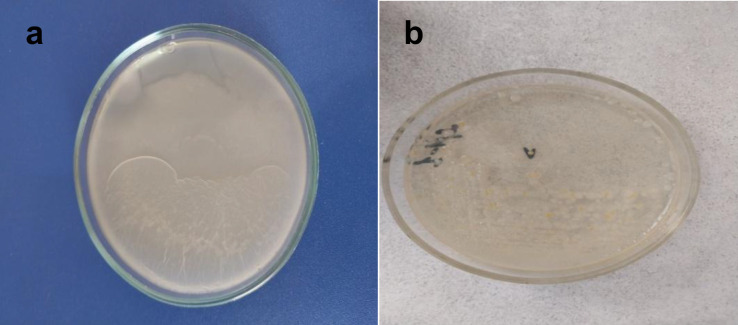
Morphological characteristics of bacterial colony growth on meat peptone agar. (a) Sample 5, incubated at 30 ± 1°C for 18h under aerobic setting; (b) Sample 2, incubated at 30 ± 1°C for 24h under aerobic setting.

On MPA, several colony morphologies were observed: beige-gray, shiny, flat, small, round colonies with smooth or irregular edges; beige-gray, matte, flat colonies with wrinkled surfaces forming confluent growth; gray colonies with a glossy, smooth surface; and occasionally yellow colonies with smooth surfaces, regular edges, and a shiny appearance (Fig 16).

**Fig 17:**
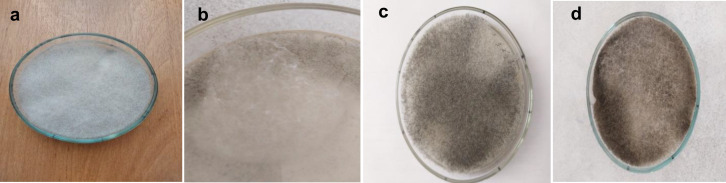
Dynamics of fungal colony growth on sabouraud agar on sample 6. Samples were incubated at 25 ± 1°C for 120 h (a), −144 h (b), 168 h (c), 192 h (d).

On Sabouraud agar (Fig 17), minimal confluent growth was observed on the first day of incubation, characterized by white colonies with a faint, fine, cotton-like aerial mycelium and black speckles (sporangia). By the third day, colony bases exhibited a yellowish coloration matching the medium, transitioning into a white aerial mycelium with dark inclusions at the tips. The colony base appeared mucilaginous and penetrated into the medium.

On days 5–7, the mycelium developed more pronounced dark speckles, turning gray-black in appearance. By day 10, the colonies became distinctly darker. This progressive darkening with age is attributed to the accumulation of sporangia, resulting in a blackish-brown coloration.

**Fig 18:**
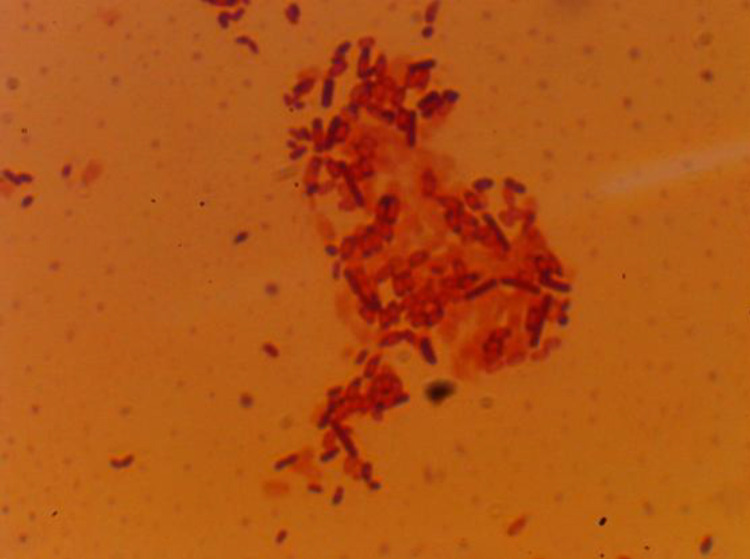
Microscopic image of Gram-stained smear, 1000 × magnification (sample 5). Germination time and sample numbers correspond to Table 3.

In Gram-stained smears prepared from MPA cultures (Fig 18), all four morphological colony types revealed the presence of Gram-positive rods with rounded ends. These cells were arranged singly, in pairs, short chains, or in dense clusters. Vegetative forms of bacteria were also observed. Gray-beige colonies exhibited centrally and subterminally located spores, while yellow colonies showed terminal and subterminal spore placement. The spores were spherical in shape.

All bacterial isolates belonged to the genus *Bacillus* sp., characterized by Gram-positive, motile rod-shaped cells with spherical spores located in various positions and exhibiting catalase-positive activity. The identification of micromycete species was performed using established taxonomic keys [37,38].

**Fig 19:**
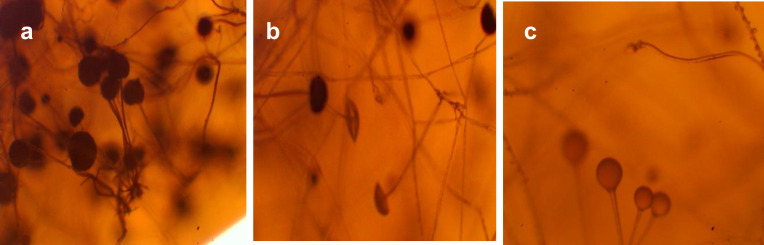
Microscopy of filamentous fungi in petri dishes, 40 × magnification (sample 15). Germination time and sample numbers correspond to Table 3. Samples were incubated at 25 ± 1°C for 120 h (a), −144 h (b), 168 h (c).

In describing the cultural characteristics of fungal colonies, the following features were assessed: colony color and mycelial morphology, shape, size, elevation profile, translucency, structural pattern, consistency, and surface texture of fungal growth on Petri dishes (Fig 19).

The image shows a mold fungus belonging to the genus *Rhizopus*, order *Mucorales*, family *Rhizopodaceae*. The darker the mycelium, the darker the background in the image. The hyphae are long, thin, and intricately interwoven. The sporangiophores are light brown, unbranched, and arranged in clusters of 3–5 at nodes opposite the rhizoids. At the tips of the sporangiophores are spherical or hemispherical sporangia, which gradually darken as they mature. These structures contain developing spores. The released spores vary in size and exhibit irregular shapes—trapezoidal, angular, spherical, or ovoid—and range in color from brown to black.

**Fig 20:**
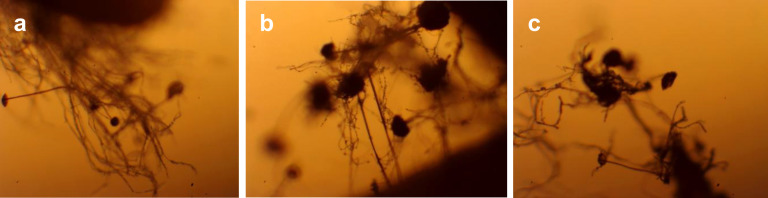
Microscopy of wheat grain at 40 × magnification (sample 15): (a) control grain, (b) germinated grain, (c) germinated sprout. Germination time and sample numbers correspond to Table 3.

Microscopic analysis of both control and germinated wheat grain samples (Fig 20) confirmed the presence of fungal cultures. Filamentous fungi were observed in both the control and germinated grains.

Thus, the dynamic analysis of wheat grain germination revealed a characteristic pattern of microbial growth typical for cereal crops, with the exception that no yeast fungi were detected. The microflora composition of *T*ә*uelsizdik* wheat grain was represented by aerobic spore-forming bacteria (*Bacillus* spp.) and filamentous fungi (*Rhizopus* spp.).

### SEM imaging of germinated wheat grain

**Fig 21:**
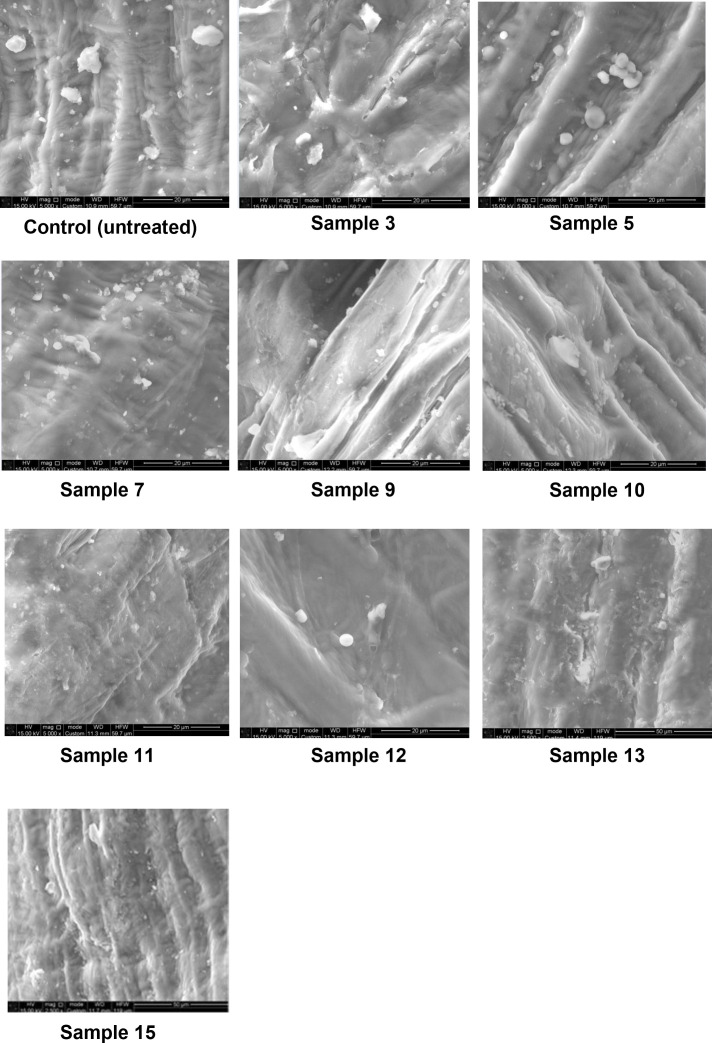
Scanning electron micrographs of wheat grain in the initial state and after germination under various conditions. Germination time and sample numbers correspond to Table 3.

The obtained SEM images of cross-sections of wheat grains provide detailed insight into structural changes occurring during germination (Fig 21). The cross-sectional analysis revealed a transformation of starch granules from well-defined, large, and regularly shaped structures in the initial sample to smaller, irregularly shaped granules as germination progressed.

### Raman spectroscopic analysis of germinated wheat grain

**Fig 22:**
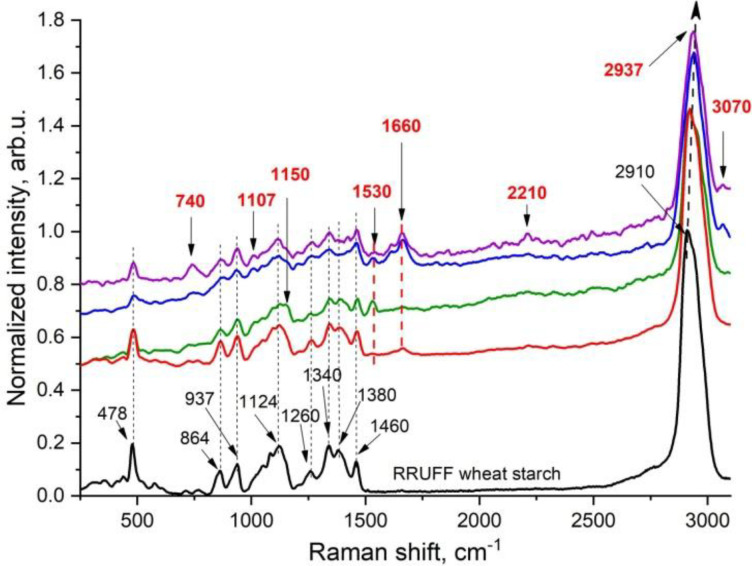
Raman spectra of the control wheat grain sample.

Raman spectra of the samples exhibit peaks that closely correspond to those characteristic of starch, observed at 478, 864, 937, 1124, 1260, 1340, 1380, and 1460 cm ⁻ ¹, as well as at 2920 and 2950 cm ⁻ ¹. Minor shifts of up to 4 cm ⁻ ¹ are acceptable due to the instrument’s spectral resolution. The disappearance, broadening, or reduced intensity of these peaks indicates the degradation of starch. Additionally, certain regions of the grain display the emergence of peaks not associated with starch. A review of the literature suggests that peaks around 740, 1150, and 1530 cm ⁻ ¹ are attributable to carotenoid functional groups [39–41]. The peak near 1660 cm ⁻ ¹ and its shoulder at approximately 1610 cm ⁻ ¹ are associated with the amide I band and C = C bonds, respectively—the latter being characteristic of lignin. Furthermore, in some samples, an additional peak around 3070 cm ⁻ ¹ was observed, which corresponds to C–H bonds typically found in terpenoids. The appearance of a peak near 1007 cm ⁻ ¹ is likely related to the presence of phytic acid (Fig 22).

The typical spectra of the initial wheat grain sample, along with the reference spectrum of wheat starch from the RRUFF database [42] for comparison, demonstrated that the primary peaks of the sample closely matched those of starch at 478, 864, 937, 1124, 1260, 1340, 1380, and 1460 cm ⁻ ¹.

Thus, according to the Raman spectroscopy data, starch is the primary (dominant) component present in all samples, as evidenced by the characteristic peaks. Deviations from the typical starch signal were observed in localized areas of the grain and were consistent across all samples—that is, the appearance or disappearance of certain peaks did not vary significantly between samples.

### Optical microscopy of wheat grain during germination

Optical microscopy images of wheat grain in its initial state and after germination under various conditions show that the dense, vitreous structure of the native grain gradually becomes porous and dull as germination progresses (Fig 24).

**Fig 23:**
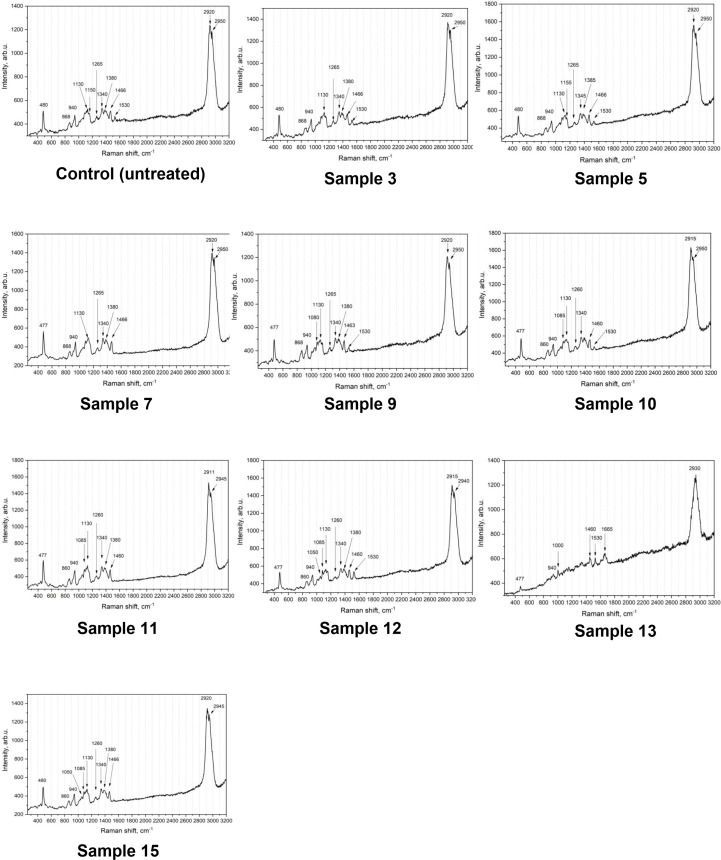
Raman spectroscopy data of control and germinated soft wheat grain under various conditions.

**Fig 24:**
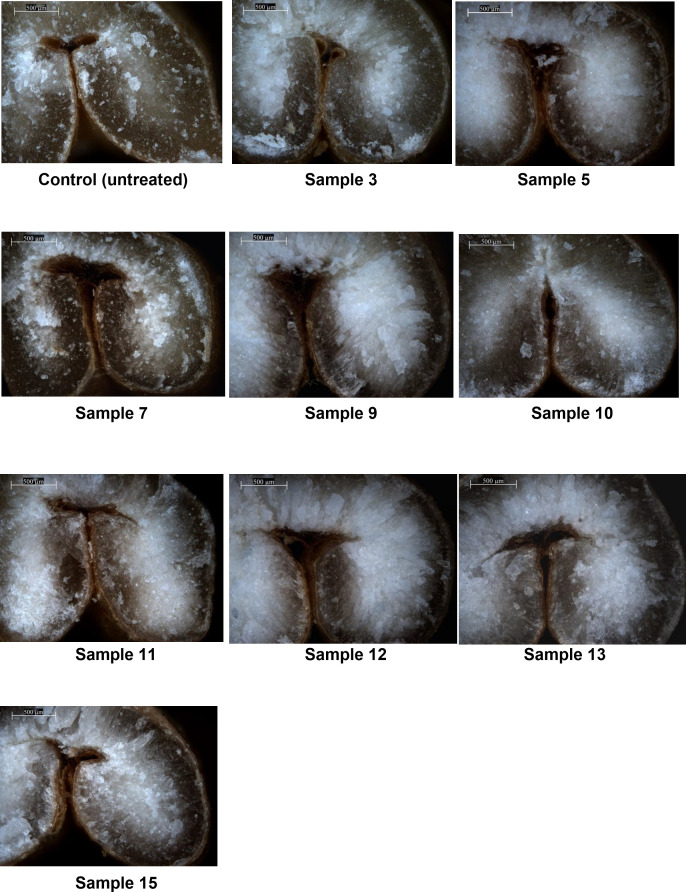
Optical micrographs of control and germinated grain under various conditions. Germination time and sample numbers correspond to Table 3.

## Discussion

The alterations induced by the germination of soft wheat grain particularly within its starchy component represent a key factor influencing the industrial applicability of germinated wheat [20]. Soft wheat cultivated in Kazakhstan, grown under increasingly stressful climatic conditions similar to those observed globally, faces significant challenges in terms of utilization once germination has occurred [43]. The findings of the present study provided systematic insights and novel perspectives on the microstructural and microbiological changes in germinated wheat grain, offering valuable information for advancing its potential applications in food and feed industries.

The physical properties of germinated wheat grain are directly influenced by the germination conditions. As germination time increases, α-amylase activity rises, leading to an exponential decrease in the Falling Number. According to Hull, Swanepoel [5], even a slight increase in α-amylase activity can cause a significant reduction in the Falling Number. This trend was also observed in our study, where a 72-hour germination period resulted in a 74% decrease in the Falling Number. Li, Pan [44] suggest that extremely high α-amylase activity, reflected by a reduced Falling Number in mature wheat grain, often leads to quality deterioration and a sharp decline in market value.

Germination involves growth and respiration processes, contributing to dry matter loss [5]. They also attributed yield loss due to pre-harvest sprouting to endosperm degradation, which reduces test weight and causes substantial economic losses. Karim and Mahmoud [45] also linked dry matter loss to microbial activity. While Krapf, Ding [46] reported a maximum 5% dry matter loss over 3 days of germination, the current study observed an 8.2% decrease in thousand-kernel weight and a 22% reduction in test weight over 72 hours. Previous study documented up to 15% loss during germination over three days at 20–30°C [46]. These pronounced declines in physical parameters confirm significant quantitative losses during pre-harvest sprouting—posing serious concerns for wheat producers. Krapf, Ding [46] established temperature as a critical factor influencing α-amylase activity and dry matter loss during germination.

α-Amylase is one of the key enzymes involved in starch degradation during germination, hydrolyzing α-1,4-glycosidic bonds and converting starch into dextrins, maltose, and glucose [46]. In germinated grains, amylases catalyze the hydrolysis of amylose and amylopectin into simple reducing sugars (glucose and maltose), and to a lesser extent, non-reducing sugars like sucrose [20,47]. In contrast to Ahmed, Ragab [27], who reported an increase in starch content during germination, Kaur, Gasparre [24] observed a reduction. The latter identified starch as the most variable component during germination, with reductions occurring as early as 36 or 72 hours depending on experimental conditions. In our study, starch content increased in the initial grain from 57.48% to 68.62% after 72 hours at 25°C and 18.0% moisture—an overall 17% increase. We hypothesize that the pattern and rate of starch changes are directly determined by the initial quality characteristics of the grain. Therefore, establishing consistent dependencies requires broader investigations involving a larger number of soft wheat grain samples with varying initial quality parameters.

Protein content also showed inconsistent patterns. While Kaur, Gasparre [24] and Muñoz-Llandes, Martínez-Villaluenga [20] found no significant effect of germination or temperature on protein levels. Others reported increased protein content, likely due to enzymatic degradation and the release of free nitrogen [48,49]. In our study, protein content generally decreased (up to −7.1%), although under high temperature (30–33°C) and extended germination (experiments 9, 11, 13), stability or minor increases (up to +0.2%) were observed, with the largest decline reaching 12.0%. The differences observed in the results may be attributed to several factors: the degree of protein hydrolysis during germination, varietal characteristics, and the methods used for protein determination (total nitrogen content versus protein fraction analysis). During germination, a portion of storage proteins is hydrolyzed into amino acids and low-molecular-weight peptides, which can lead to a reduction in measurable total protein content when using conventional methods such as the Kjeldahl method [50]. Proteomic studies confirm the degradation of high-molecular-weight proteins during germination [51]. However, with prolonged germination and elevated temperatures, the accumulation of stable nitrogen-containing compounds may occur, partially compensating for these losses [52].

Studies have demonstrated that microbial contamination of sprouts can originate from multiple sources before and after harvest, including seed material, germination substrates, soaking water, as well as handling, transportation, and storage conditions [47]. In line with these findings, our study showed that the initial grain sample contained bacterial loads of approximately 10 × 10⁶ CFU/g and filamentous fungi at levels around 10 × 10¹¹ CFU/g, which are consistent with previously reported data. For instance, Badawy, Al-Dalain [48] noted that wheat used for processing typically contained bacterial counts ranging from 3 to 8 log CFU/g and yeast and mold counts from 2 to 7 log CFU/g, equivalent to 10³–10⁸ CFU/g and 10²–10⁷ CFU/g, respectively. Similarly, Manthey, Wolf-Hall [53] reported microbial loads of 8.2 ± 1.3 log CFU/g in durum wheat harvested in 2001, corresponding to approximately 10⁹ CFU/g. According to Krapf, Ding [46], microbial growth doubles with every 10°C temperature increase until a thermal threshold is reached. Acceptable microbial levels were maintained during germination for up to 4 days at 15–20°C, but higher temperatures or extended germination caused a sharp rise in aerobic bacteria and molds. Fungal presence was detected after 4 days at 25°C or 8 days at 15°C. This was confirmed in our study, where bacterial counts increased by 5–6 orders of magnitude, reaching 10¹⁰ CFU/g. Fungal loads also reached 10¹⁰ CFU/g.

Microstructural changes are of particular importance, as they reflect internal degradation affecting wheat’s processing potential. According to Muñoz-Llandes, Martínez-Villaluenga [20], starch granule morphology changes during germination, with enzyme-induced pores and surface damage diminishing the granules’ structural integrity and water-holding capacity. Germination (35°C, 48 h) of brown rice caused visible surface erosion and reduced granule size. Our SEM analysis confirmed such changes in wheat grain. The control sample exhibited dense starch–protein matrices with intact networks (Fig 22). During soaking, grains absorbed water, swelled, and triggered enzymatic activity. This resulted in a looser microstructure, making starch granules more distinct (samples 9, 12). At longer germination periods, degradation of starch and protein networks became pronounced (samples 13, 15), highlighting extensive enzymatic restructuring and reserve mobilization [54].

Optical microscopy confirmed the structural alterations in wheat grain during germination—an initially dense, vitreous structure in the ungerminated control sample (Fig 24, initial grain) progressively transformed into a loose and dull morphology as germination advanced (Fig 24, samples 3, 5, 7, 9–13, 15). This is likely caused by enzymatic breakdown of hemicellulose and cellulose in cell walls [9]. Furthermore, two-dimensional cross-sectional analyses revealed the formation of pores within the grain structure. While no internal porosity was observed in the ungerminated control sample (Fig 21, initial grain), pronounced pore development became evident as early as 24 hours into germination (Fig 21, samples 3 and 5), indicating structural degradation associated with elevated α-amylase activity. The presence of pores in the cross-sections of samples germinated for 24 hours or more suggests several phenomena: increased tissue volume, weakening of the endosperm matrix due to enzymatic activity, and the initial breakdown of cell walls—hallmarks of germination. Additionally, the appearance of white patches during germination is indicative of tissue hydration and water infiltration [9,55,56].

Raman spectroscopy enables the chemical and structural characterization of major cereal grain components by analyzing longitudinally bisected kernels [57]. In the present study, this technique confirmed the degradation of starch in wheat grains during germination. Deviations from the characteristic starch spectral profile were observed in localized regions across all samples. These variations, such as the appearance or disappearance of specific peaks were consistent in nature and did not significantly differ between individual samples. According to Sanchez, Ermolenkov [58], variations in the intensity of lipid-associated Raman peaks (2920 and 2950 cm ⁻ ¹) and those linked to carbohydrates (approximately 940 and 1130 cm ⁻ ¹) are indicative of active starch hydrolysis into simple sugars during germination. The emergence and amplification of amide and protein-associated peaks (1340–1530 cm ⁻ ¹) may reflect enhanced enzymatic activity and the synthesis of new proteins. Additionally, an increase in the peak near 1265 cm ⁻ ¹ may be associated with the activation of enzymes involved in germination processes. A similar spectral pattern was observed in our study. As illustrated in Fig 23, the peaks corresponding to lipid fractions remained relatively stable, ranging from 2911 to 2940 cm ⁻ ¹. Minor changes were detected in the amide and protein regions (1340–1530 cm ⁻ ¹), while the most pronounced spectral fluctuations were observed in the carbohydrate region (940–1340 cm ⁻ ¹). These findings highlight the need for further Raman spectroscopic investigations of germinated wheat grains under varying germination conditions, supported by specialized sample preparation techniques such as microtoming to obtain thin sections or milling followed by pellet formation to enhance spectral resolution and analytical accuracy.

## Conclusion

Soft wheat grain undergoes significant physical and chemical changes under varying germination conditions. The reduction in dry matter observed may result in considerable quantitative losses at harvest on a national scale. The study identified the optimal parameters for the germination of soft wheat grain. The optimal range for maximizing starch content was achieved at a moisture level of 22%, a temperature of 31°C, and a germination time of 84 hours. The optimal conditions for maximizing protein content were determined as a moisture level of 21%, a temperature of 30°C, and a germination time of 72 hours. Analysis of the response surface behavior indicated that the optimal parameters for minimizing the total microbial contamination of the flour were a moisture level of 14%, a temperature of 33°C, and a germination time of 8 hours.

Chemical transformations during germination occur unevenly and are strongly dependent on temperature, moisture, and time. Extended germination alters starch granule morphology, creating surface dents. This highlights the potential of germination-induced starch modification as a sustainable alternative to chemical modification for industrial applications. Further research is warranted to define optimal germination parameters for producing starches with tailored microstructural and functional characteristics. The findings of this study provide valuable information to support the broader utilization of germinated soft wheat grain in food and non-food industries.

## Supporting information

S1 Fig Visualization of seed condition. (ZIP)

S1 Table The regression equations for protein and microbial contamination. (DOCX)
