# Supplementary figures and images for: Physicochemical, microbiological, and microstructural changes in germinated wheat grain
Source: PLoS One. 2025 Sep 9;20(9):e0331620. doi: 10.1371/journal.pone.0331620 (PMC12419666; doi:10.1371/journal.pone.0331620)

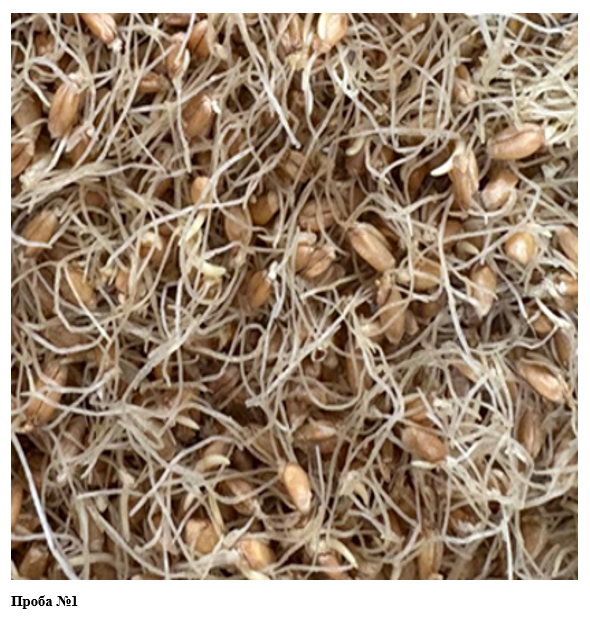

Supplement: S1 Fig — (ZIP) [file pone.0331620.s001.zip › S1 Fig/1.png]

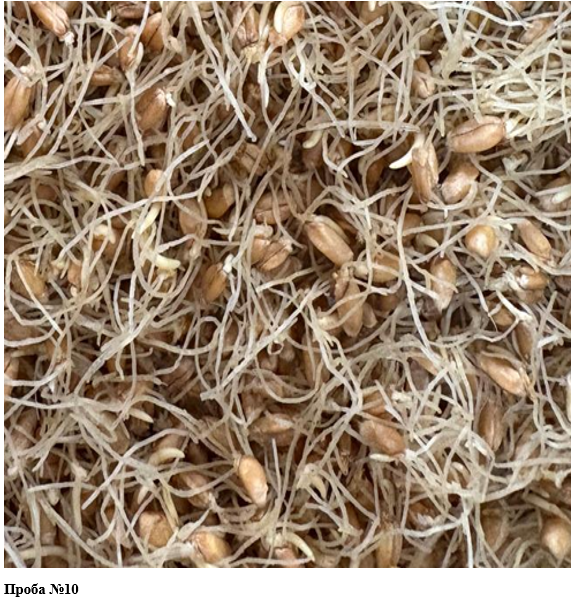

Supplement: S1 Fig — (ZIP) [file pone.0331620.s001.zip › S1 Fig/10.png]

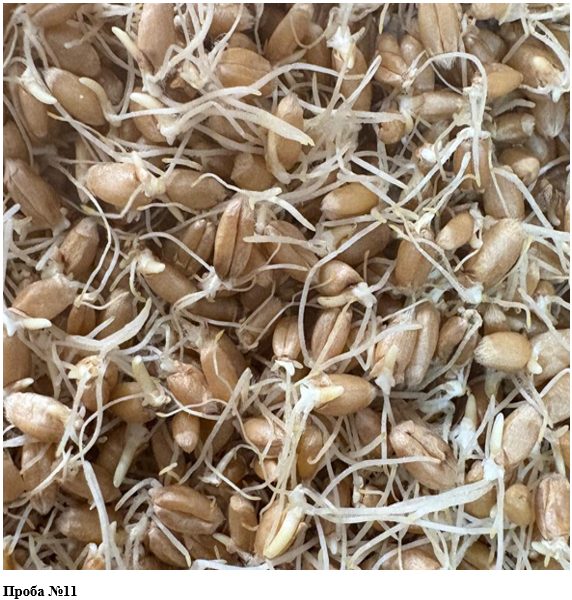

Supplement: S1 Fig — (ZIP) [file pone.0331620.s001.zip › S1 Fig/11.png]

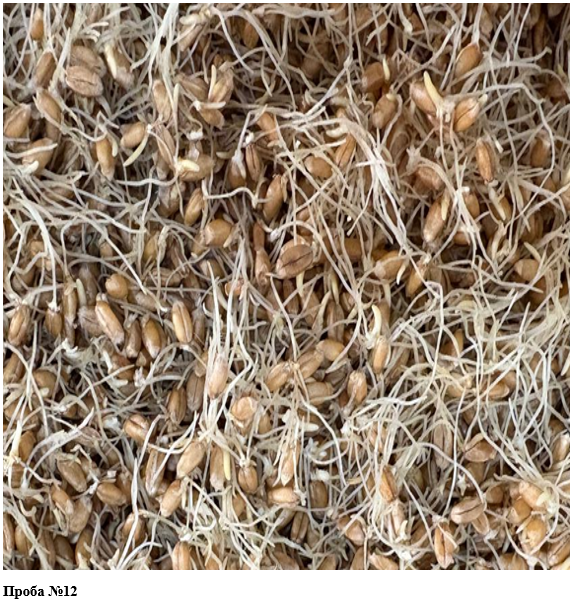

Supplement: S1 Fig — (ZIP) [file pone.0331620.s001.zip › S1 Fig/12.png]

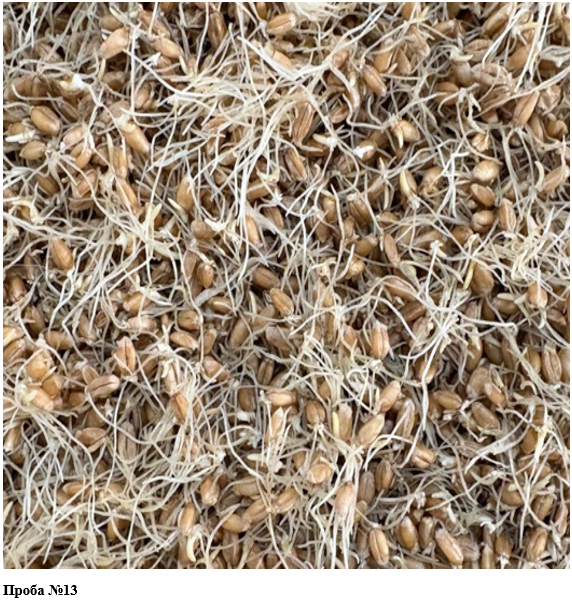

Supplement: S1 Fig — (ZIP) [file pone.0331620.s001.zip › S1 Fig/13.png]

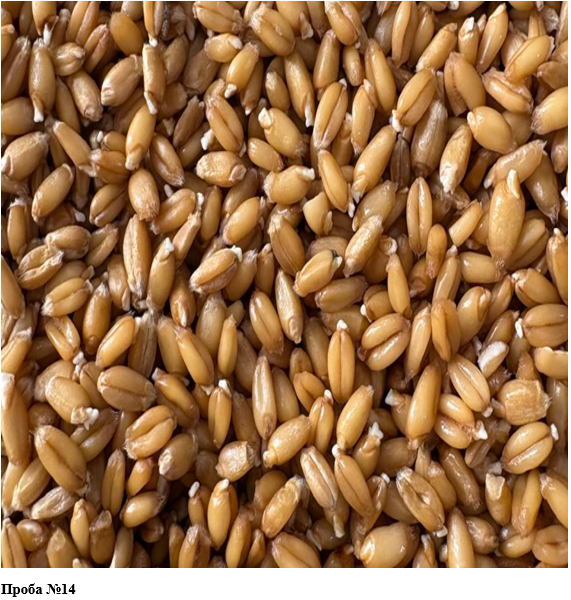

Supplement: S1 Fig — (ZIP) [file pone.0331620.s001.zip › S1 Fig/14.png]

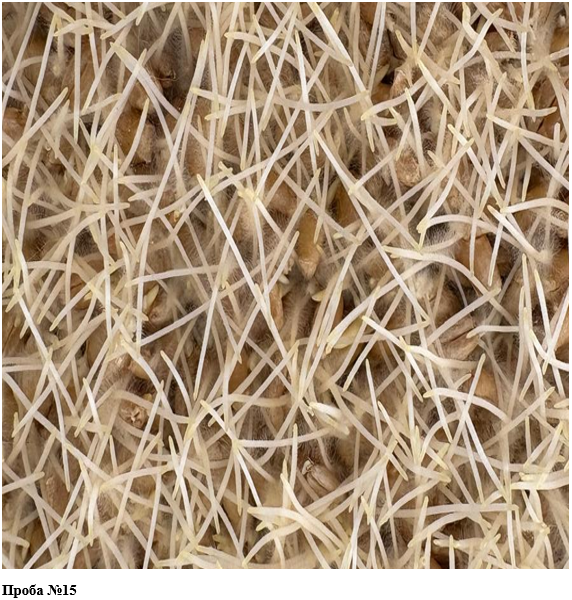

Supplement: S1 Fig — (ZIP) [file pone.0331620.s001.zip › S1 Fig/15.png]

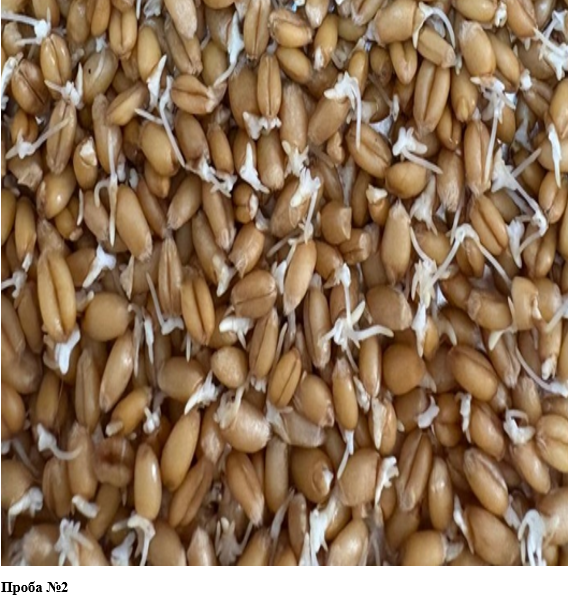

Supplement: S1 Fig — (ZIP) [file pone.0331620.s001.zip › S1 Fig/2.png]

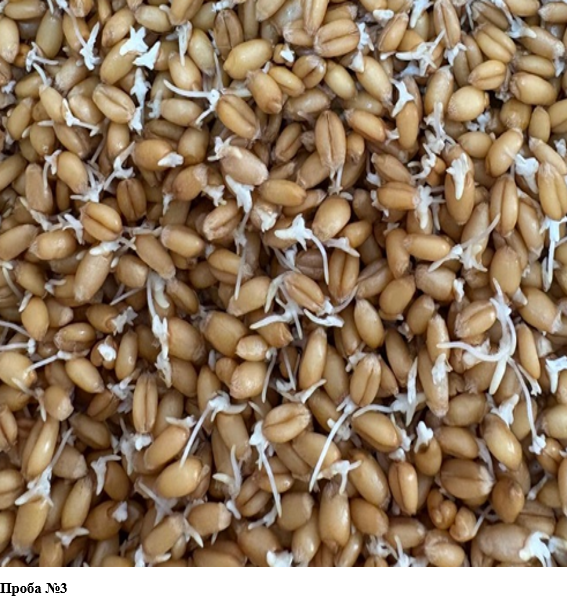

Supplement: S1 Fig — (ZIP) [file pone.0331620.s001.zip › S1 Fig/3.png]

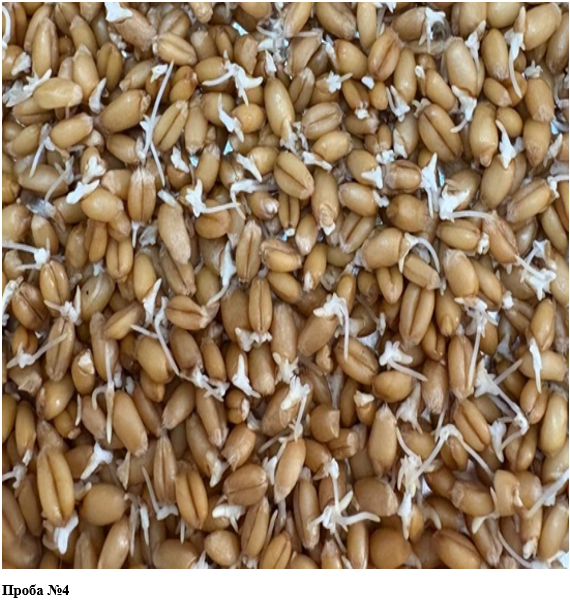

Supplement: S1 Fig — (ZIP) [file pone.0331620.s001.zip › S1 Fig/4.png]

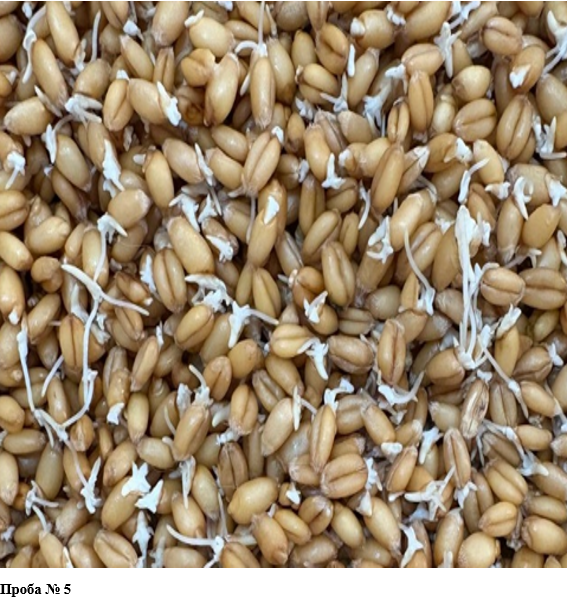

Supplement: S1 Fig — (ZIP) [file pone.0331620.s001.zip › S1 Fig/5.png]

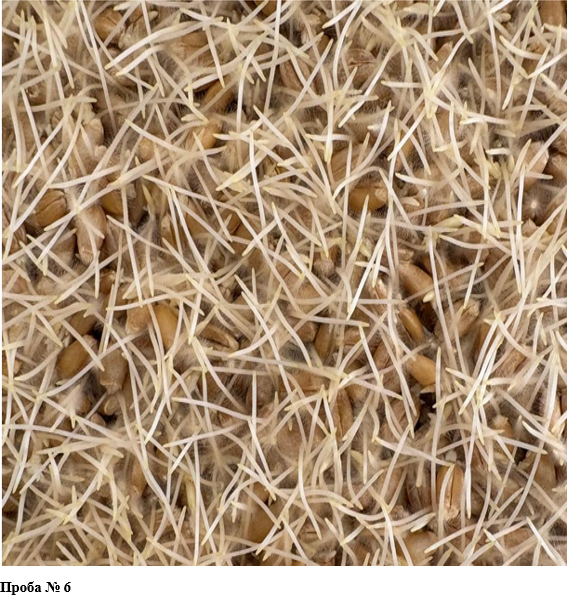

Supplement: S1 Fig — (ZIP) [file pone.0331620.s001.zip › S1 Fig/6.png]

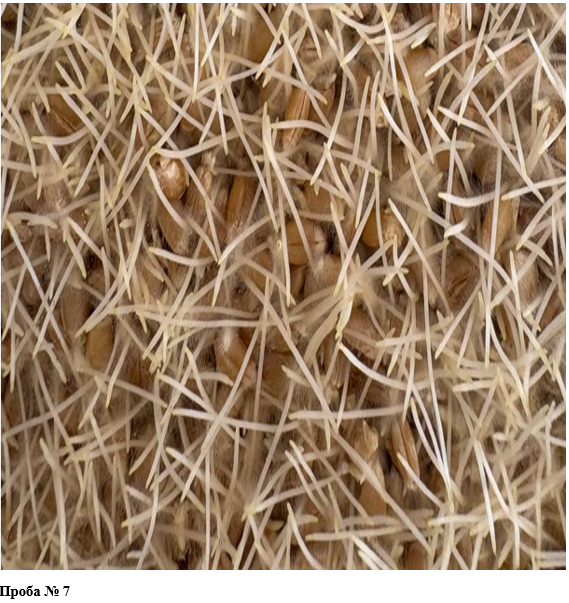

Supplement: S1 Fig — (ZIP) [file pone.0331620.s001.zip › S1 Fig/7.png]

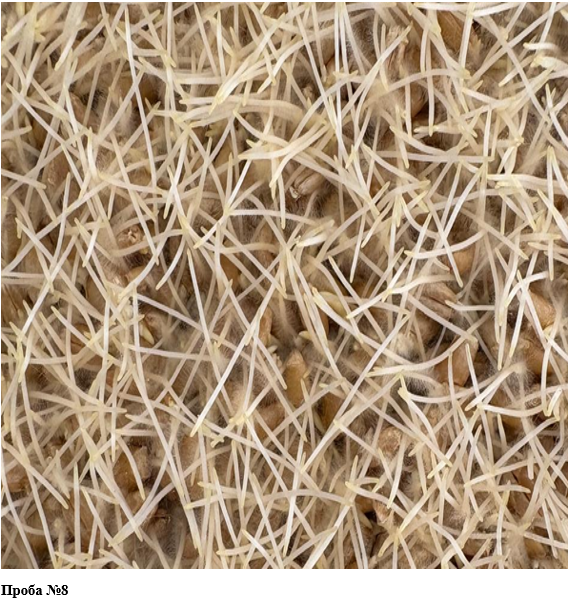

Supplement: S1 Fig — (ZIP) [file pone.0331620.s001.zip › S1 Fig/8.png]

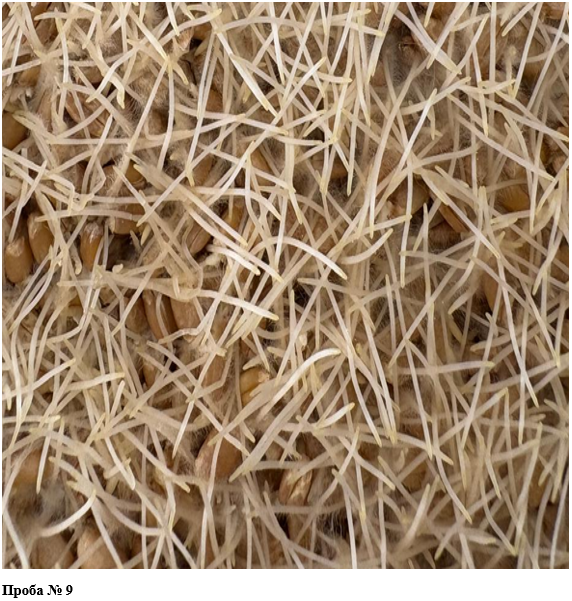

Supplement: S1 Fig — (ZIP) [file pone.0331620.s001.zip › S1 Fig/9.png]
