# Supplementary material for: Physicochemical, microbiological, and microstructural changes in germinated wheat grain
Source: PLoS One. 2025 Sep 9;20(9):e0331620. doi: 10.1371/journal.pone.0331620 (PMC12419666; doi:10.1371/journal.pone.0331620)
Supplement: S1 Table — (DOCX) [file pone.0331620.s002.docx]

The results of the model fitting for starch mass fraction and the regression coefficients are presented in Table 1.

Table 1 – Regression Coefficients

| Coefficients | Values |
| --- | --- |
| Constant | 46,9618 |
| *х_1_* | 0,982597 |
| *х_2_* | 0,587551 |
| *х_3_* | 0,0534429 |
| *х_1_^2^* | -0,0233339 |
| *х_1_х_2_* | 0,0033 |
| *х_1_х_3_* | 0,0003125 |
| *х_2_^2^* | -0,0106418 |
| *х_2_х_3_* | -0,000135417 |
| *х_3_^2^* | -0,000320709 |

Subsequently, an analysis of variance (ANOVA) for the protein content was performed, and the results are presented in Table 2.

**Table 2 – Analysis of Variance (ANOVA) for Protein Content**

| Values | Sum of Squares | Degrees of Freedom (df) | Mean Square | F-Ratio | p-Value |
| --- | --- | --- | --- | --- | --- |
| *х_1_* | 1,20454 | 1 | 1,20454 | 19,99 | 0,0042 |
| *х_2_* | 1,23661 | 1 | 1,23661 | 20,53 | 0,0040 |
| *х_3_* | 1,19027 | 1 | 1,19027 | 19,76 | 0,0044 |
| *х_1_^2^* | 0,179255 | 1 | 0,179255 | 2,98 | 0,1353 |
| *х_1_х_2_* | 0,0 | 1 | 0,0 | 0,00 | 1,0000 |
| *х_1_х_3_* | 0,0 | 1 | 0,0 | 0,00 | 1,0000 |
| *х_2_^2^* | 0,179255 | 1 | 0,179255 | 2,98 | 0,1353 |
| *х_2_х_3_* | 0,00045 | 1 | 0,00045 | 0,01 | 0,9339 |
| *х_3_^2^* | 0,183841 | 1 | 0,183841 | 3,05 | 0,1313 |
| Residual | 0,361462 | 6 | 0,0602437 |  |  |
| Total | 4,28779 | 15 |  |  |  |

The results of the model fitting for the protein mass fraction and the regression coefficients are presented in Table 3.

**Table 3 – Regression Coefficients**

| Coefficients | Values |
| --- | --- |
| Constant | -1,28697 |
| *х_1_* | 0,920016 |
| *х_2_* | 0,341384 |
| *х_3_* | 0,0373418 |
| *х_1_^2^* | -0,0222562 |
| *х_2_^2^* | -0,00556404 |
| *х_2_х_3_* | -0,0000625 |
| *х_3_^2^* | -0,000244567 |

At the next stage of the study, an analysis of variance (ANOVA) for total microbial contamination was conducted, and the results are presented in Table 4.

**Table 4 – Analysis of Variance (ANOVA) for Total Microbial Contamination**

| Values | Sum of Squares | Degrees of Freedom (df) | Mean Square | F-Ratio | p-Value |
| --- | --- | --- | --- | --- | --- |
| *х_1_* | 8,79123E19 | 1 | 8,79123E19 | 12,14 | 0,0131 |
| *х_2_* | 1,67757E17 | 1 | 1,67757E17 | 0,02 | 0,8840 |
| *х_3_* | 139264, | 1 | 139264, | 0,00 | 1,0000 |
| *х_1_^2^* | 2,40849E19 | 1 | 2,40849E19 | 3,33 | 0,1180 |
| *х_1_х_2_* | 4,05E19 | 1 | 4,05E19 | 5,59 | 0,0559 |
| *х_1_х_3_* | 4,05E19 | 1 | 4,05E19 | 5,59 | 0,0559 |
| *х_2_^2^* | 9,50649E19 | 1 | 9,50649E19 | 13,13 | 0,0111 |
| *х_2_х_3_* | 0,0 | 1 | 0,0 | 0,00 | 1,0000 |
| *х_3_^2^* | 1,75654E17 | 1 | 1,75654E17 | 0,02 | 0,8813 |
| Residual | 4,34416E19 | 6 | 7,24026E18 |  |  |
| Total | 3,3858E20 | 15 |  |  |  |

The results of the model fitting for total microbial contamination and the regression coefficients are presented in Table 5.

**Table 5 – Regression Coefficients**

| Coefficients | Values |
| --- | --- |
| Constant | -1,22×10^11^ |
| *х_1_* | 7,6×10^9^ |
| *х_2_* | 3,14×10^9^ |
| *х_3_* | 6,52×10^8^ |
| *х_1_^2^* | -2,58×10^8^ |
| *х_1_х_2_* | 1,8×10^8^ |
| *х_1_х_3_* | -3,75×10^7^ |
| *х_2_^2^* | -1,28×10^8^ |
| *х_2_х_3_* | 1,53×10^-5^ |
| *х_3_^2^* | 2,39*10^5^ |

Table 6 – Optimization of the Soft Wheat Grain Germination Process Based on the Desirability Function

| Factors | Min. | Max. | Optimum | Response | Optimum |
| --- | --- | --- | --- | --- | --- |
| Moisture Content, % | 13,8 | 22,2 | 17,5 | Starch Mass Fraction, % | 69,0 |
| Temperature, °C | 16,6 | 33,4 | 33,4 | Protein Mass Fraction, % | 14,5 |
| Time, h | 7,6 | 88,4 | 72,3 | Total Microbial Contamination, CFU/g | 1010 |

Thus, the obtained results made it possible to determine the optimal parameters for soft wheat grain germination through the application of the developed mathematical model.
